# Dual Model Medical Invoices Recognition

**DOI:** 10.3390/s19204370

**Published:** 2019-10-10

**Authors:** Fei Yi, Yi-Fei Zhao, Guan-Qun Sheng, Kai Xie, Chang Wen, Xin-Gong Tang, Xuan Qi

**Affiliations:** 1 Key Laboratory of Exploration Technologies for Oil and Gas Resources, Yangtze University, Ministry of Education, Wuhan 430100, China; hkhk900@163.com (F.Y.); tangxingong@163.com (X.-G.T.); 2 School of Electronic Information, Yangtze University, Jingzhou 434023, China; yifei666666@hotmail.com (Y.-F.Z.); 500646@yangtzeu.edu.cn (K.X.); 3 School of Computer Science, Yangtze University, Jingzhou 434023, China; 400100@yangtzeu.edu.cn; 4 School of Petroleum Engineering, China University of Petroleum, Beijing 102249, China; 2017212184@student.cup.edu.cn

**Keywords:** medical invoices, breakpoint font, CNN, RNN, semantic revisions

## Abstract

Hospitals need to invest a lot of manpower to manually input the contents of medical invoices (nearly 300,000,000 medical invoices a year) into the medical system. In order to help the hospital save money and stabilize work efficiency, this paper designed a system to complete the complicated work using a Gaussian blur and smoothing–convolutional neural network combined with a recurrent neural network (GBS-CR) method. Gaussian blur and smoothing (GBS) is a novel preprocessing method that can fix the breakpoint font in medical invoices. The combination of convolutional neural network (CNN) and recurrent neural network (RNN) was used to raise the recognition rate of the breakpoint font in medical invoices. RNN was designed to be the semantic revision module. In the aspect of image preprocessing, Gaussian blur and smoothing were used to fix the breakpoint font. In the period of making the self-built dataset, a certain proportion of the breakpoint font (the font of breakpoint is 3, the original font is 7) was added, in this paper, so as to optimize the Alexnet–Adam–CNN (AA-CNN) model, which is more suitable for the recognition of the breakpoint font than the traditional CNN model. In terms of the identification methods, we not only adopted the optimized AA-CNN for identification, but also combined RNN to carry out the semantic revisions of the identified results of CNN, meanwhile further improving the recognition rate of the medical invoices. The experimental results show that compared with the state-of-art invoice recognition method, the method presented in this paper has an average increase of 10 to 15 percentage points in recognition rate.

## 1. Introduction

### 1.1. Detailed Introduction

A large number of paper medical invoices are produced in hospitals every day. If we only use manpower to identify and classify these medical invoices, it is not only a waste of manpower, but also cannot guarantee work efficiency after long working hours [1]. Compared to a similar field, such as for face recognition, one of the best methods is based on Gabor jet feature extraction, filters, and Borda count classification [2,3,4,5], which really point out that the faster the image features are processed, the better the recognition they will present. In order to improve and stabilize the recognition efficiency of the medical invoices, we made a brand-new solution to deal with the problem facing the whole medical system.

From the inchoate object recognition [6], we found that the recognition field became more and more specific, as with face recognition [7,8,9], text recognition [10,11,12], and speech recognition [13,14]. In the text recognition area, there are some ways to achieve the recognition of traditional invoices, such as the bank bill automation recognition based on the HMMs (hidden Markov models) method, invented by Gui-Xin Wang [15] and recognition algorithms on bank invoice images with a BP (back propagation) network, invented by Meng-di Han [16]. However, there is still a blank space for Chinese medical invoices. Meanwhile, the traditional recognition methods could not achieve the goal of making the whole process both efficient and reliant. Recently, image representations based on the convolutional neural network (CNN) have attracted increasing interest in the community, and have demonstrated an impressive performance [16], for example for Chinese handwriting recognition. As for the medical area, CNN has been used in medical images processing [17], and recurrent neural network (RNN) has been applied to solve the problem of language semantics [18]. The combination of CNN and RNN has already been used in dealing with the recognition of handwritten English words [19], atmospheric visibility from images [20], image visual recognition [21], and action capture [22]. It shows that the mature framework could work in all kinds of image problems, including single layer and multiple layer images [23].

However, the combination of CNN and RNN has not been used in the area of invoice recognition. A paper by D. Lowe proposed a method for object recognition using distinctive image features—it extracts the distinctive invariant features from the images that can be used to perform reliable matching between different views of an object or scene. The features are invariant to the image scale and rotation, and at the same time they are shown to provide robust matching across a substantial range of affine distortions, change in 3D viewpoints, addition of noise, and change in illumination. The features are highly distinctive, in the sense that a single feature can be correctly matched with a high probability against a large database of features from many images [6]. This really inspired us in the field of dealing with image feature processing. We came up with a way of combining artificial intelligence, image preprocessing, deep learning, and CNN, along with RNN, as a new network model to deal with this problem. In our project, we firstly used image grayscale and image binarization to extract the features, then, we used the Gaussian blur and smoothing to fix the breakpoint font. We named this image preprocessing method Gaussian blur and smoothing (GBS). We figured out a special connection between the word datasets and the words we were trying to recognize by using a deep learning training model developed by CNN, and built up another training model based on RNN about the meanings of the words that we recognized from the CNN. To activate the features we got by image preprocessing so as to match the datasets, we used a new method—using a single letter segmentation program that we built to pre-cut the continuous characters into single characters, which we then sent to our CNN and RNN network to recognize. The whole new model of the process that we made was named the GBS-CR model. It really matches the advanced leading thinking of using deep learning and image processing to upgrade the recognition technology from the traditional to advanced methods.

### 1.2. The Motivation and the Contribution of This Paper

Hospitals need to invest a lot of manpower to manually input the contents of medical invoices into computer systems. In order to help hospitals save costs in this aspect, we designed a system to complete a lot of complicated work. In cities like Shanghai, 300,000,000 medical invoices are usually produced by hospitals, based on the population of 20 million. This means that the hospitals need their employees to take up more workload to finish jobs with the invoices, in addition to other vital paperwork. The workloads with the invoices include many terms, such as the management of the medical invoices. In today’s situation, we are still using manpower to sort the invoices and our introduced system would greatly help decrease the manpower and provide more productivity. Our method is a pioneer step for combining artificial intelligence with medical system.

Because the stylus printer uses a special font (breakpoint font) in medical invoices, the recognition accuracy is lower than the normal font used in other invoices. So, this paper improved the recognition accuracy of the breakpoint font from the following two aspects:

1. In terms of preprocessing, we abandoned the traditional methods, such as the wavelet-transform, because of the worse feedback in the breakpoint font. We designed a brand-new preprocessing method called Gaussian blur and smoothing (GBS), which can effectively repair the breakpoint font and improve recognition accuracy;
2. Compared with a state-of-art text recognition system, such as optical character recognition (OCR), we used the dual model network of CNN and RNN as the recognition module in order to improve the recognition accuracy.

As a matter of fact, this system helps hospitals accomplish a large amount of complicated work.

## 2. Background

### 2.1. The Background of Deep Learning

Today, deep learning is already becoming a much-needed technology in many areas, especially in recognition and further processing. As for the deep learning technology we currently put in use, it started from the BP model, well-known as the multi-layer perceptron. It is the primary model of the deep learning methods, but it is still limited by the lack of calculation power of computers. With the development of computing power, more complex methods with better accuracy have appeared. In 2006, Professor Geoffrey Hinton and his students published a paper on science, and pointed out that in many areas, the gathering features of the datasets were more useful for describing themselves. In order to overcome the difficulty of how to train a network model of deep learning, he presented a new idea called layer-wise pre-training, and these two points are still what we are using nowadays in works on deep learning. Deep-learning methods are representation-learning methods with multiple levels of representation, obtained by composing simple but non-linear modules. These modules transform each representation at one level (starting with the raw input), into a representation at a higher and more abstract level. With the composition of enough transformations, very complex functions are formed [24]. In 2012, Professor Geoffrey Hinton and his students performed a further study using CNN to recognize images using only pixels. This is an advanced idea in image processing as well as in all areas of image recognition, solving the problem of too many features of images that need to be collected and replaced with pixels. In particular, CNN has achieved the top performance on many image-based classification tasks, as its structure is very suitable for representing the image data. For example, image classification used Chinese handwriting recognition [25], and it has been used in the field of face recognition as well. For another framework, we used RNN. The recurrent neural network is a powerful model for sequential data, which has been used widely in speech recognition for combining the multiple levels of representation that have proven so effective in deep networks, with flexible use in a long-range context [26,27,28]. The RNN also fits in the sentences semantic area [29]. A study by Cho, Kyunghyun shows that the proposed model (based on RNN) learns a semantically and syntactically meaningful representation of linguistic phrases [30]. It has also been proven to play an important role in the recognition of fast continuous Mandarin speech [31]. These areas have many familiar parts with word or text recognition; in particular, the semantic part is still a very challenging part of invoice recognition, which is why we fitted the RNN into our work smoothly.

### 2.2. The Status Quo of Invoices Recognition

So far, the deep learning algorithm has been applied to replace the original matching algorithm. As for the text recognition, it has always been a long-standing topic in computer vision [32]. Recognition methods in text are put forward, but are rarely based on deep learning. Another blank area is in the area of medical invoices recognition. Traditional invoice recognition only focusses on general invoices and bank invoices, for example, the bank bill automation recognition based on HMMs method invented by Gui-Xin Wang, the recognition algorithms of bank invoice images using a BP network invented by Meng-di Han, and the spiral recognition methodology for the recognition of Chinese bank bills [33].

For traditional recognition, it only can recognize the continuous characters where every word is printed clearly and smoothly. A lot of methods are based on OCR, but it is already out of date in the invoice recognition area. In recent years, deep learning methods have really outperformed traditional methods in the OCR research field, which proposes the idea that CNNs on large-scale annotated datasets can be efficiently transferred to other visual recognition tasks with a limited amount of training data [34], which gave us the idea of combining the deep learning methods with our dual model system.

### 2.3. The Innovation of GBS-CR

As we all know, for printed text, the main difficulty still comes from severely merged or degraded characters. Even now, the incorrect recognition of merged characters is still one of the main problems [35], both in printed letters and handwriting [36], but medical invoices are different. They not only have the problem above, but also medical invoices are mostly printed using the stylus printer. This means that there will many breakpoints reserved in every character, but at the moment, traditional recognition cannot recognize words with breakpoints very well. To perfectly solve the problem, we found that Gaussian blur and smoothing is an excellent method, by expanding the character itself while still being able to recognize it. In the field of face recognition, the Gaussian filter has been used as a tool to help deal with preprocessing and after blurring. We found a mature method named the scale-invariant feature transform (SIFT), proposed by David Lowe, which provided a solution to extract the features and re-match with the database. SIFT is a powerful set of local, invariant features or key-point descriptors for detecting local structures in different image views. The noises are blurred, but the details on edges remain unaffected in this scale space. Compared with traditional SIFT-based matching methods [37,38,39], the features extracted by AAg-SIFT [40,41,42,43] are more stable and precise [44,45,46,47]. This method has normally been seen in image recognition [48,49], and inspired us with how to preprocess the breakpoint character. In our work, we figured out a way to preprocess the image by using a Gaussian filter to process the words and to reach a point where the breakpoint was fixed smoothly and the necessary features were left over clearly. Thus, we could still extract the invariant features. We also applied the deep learning model to upgrade the speed in order to solve the problem of the low recognition rate of the traditional method.

This paper proposes a new image preprocessing method named GBS and a medical invoice recognition algorithm which combines CNN and RNN together, in order to recognize medical invoices, which greatly improves the performance of medical invoice recognition, after having studied the existing achievements in signal processing and deep learning in our laboratory [50,51,52]. In the algorithm combining the Alexnet with Adam optimization algorithm, using the Adam optimization algorithm can make the network convergence fast and reduce the loss of the advantages of network training, greatly improving the recognition rate.

## 3. Dual Model Medical Invoice Recognition Methods

**Figure 1:**
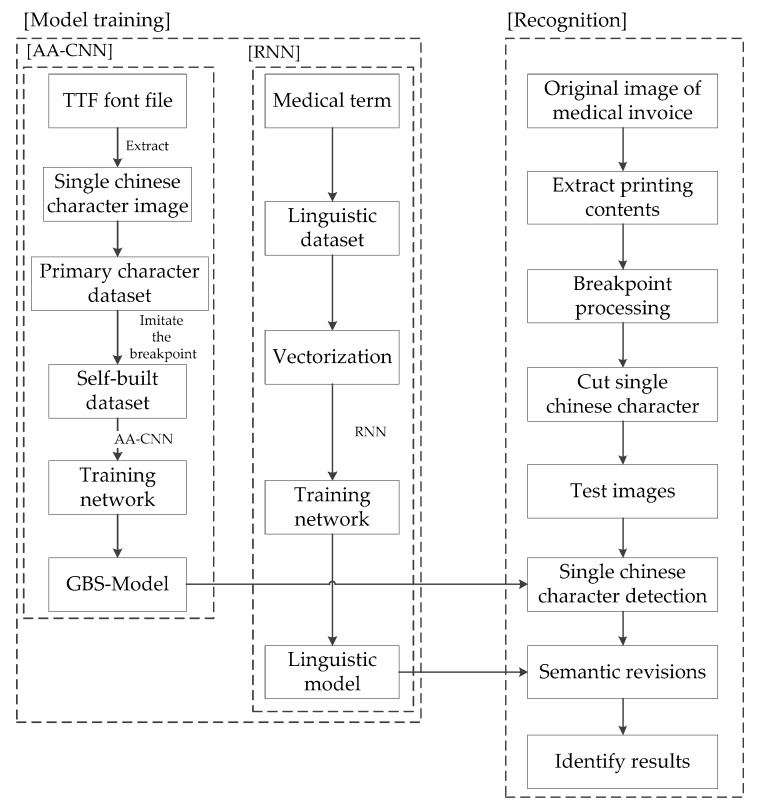
The framework of the Gaussian blur and smoothing–convolutional neural network combined with a recurrent neural network (GBS-CR). RNN—recurrent neural network.

This paper introduces the optimization and acceleration of the GBS-CR model from the following aspects, the framework of which is shown in Figure 1.

As shown in Figure 1, the GBS-CR system proposed in this paper was divided into the following two modules:

1. Model training (offline);
2. Recognition (online).

In the first module, we only needed two models trained by Alexnet-Adam-CNN and RNN. Then, we could upload these models to the cloud and save it.

In the second module, as there is a large amount of professional terminology in medical invoices, this paper combined AA-CNN and RNN in sequence. After AA-CNN outputs recognized the results, RNN was used to carry out the semantic revisions obtained by the recognized results of CNN, and modified some professional terminology in the medical invoices.

### 3.1. AA-CNN Model Training for Recognition

**Figure 2:**
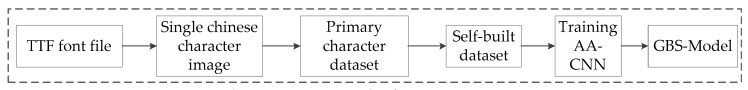
Framework of CNN processing.

After obtaining the font images of the medical invoices, we carried out the image preprocessing in order to obtain the training set required by the AA-CNN network, which integrates the Alexnet and Adam optimization algorithm, and we started network training with the primary character dataset. In the end, we could save the trained model to the local and identify the images. The specific process is shown in Figure 2.

First, we got the single Chinese character images from the TTF font file, then we put all of these images together to form a primary character dataset, which included 525,700 images from 3755 commonly used Chinese characters.

Second, because of the differences between the common invoices and medical ones, we added the appropriate percentage of simulated breakpoint fonts to build a new dataset, which included 225,300 images from 3755 commonly used Chinese characters.

**Figure 3:**
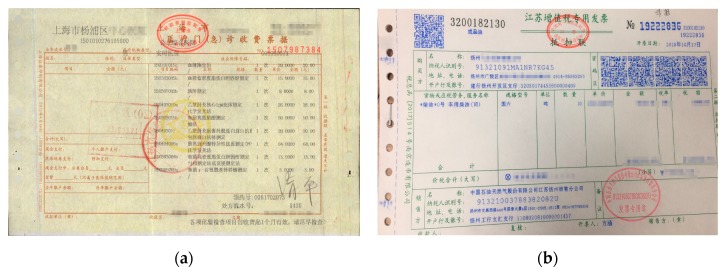
Comparison of invoices: (**a**) medical invoice; (**b**) general invoice.

In view of the different fonts between the normal notes and medical invoices, we designed a method to imitate the breakpoint font. The comparison of these two kinds of invoices is shown in Figure 3.

**Figure 4:**
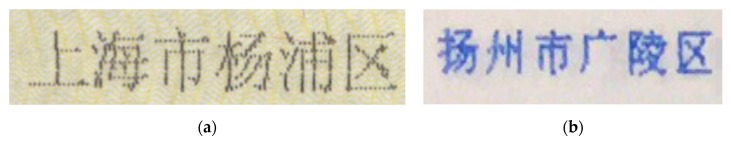
Comparison of two kinds of enlarged invoices: (**a**) medical invoice; (**b**) general invoice.

We can clearly see from Figure 4 that the font of the general invoice is continuous, while the medical one is broken.

**Figure 5:**
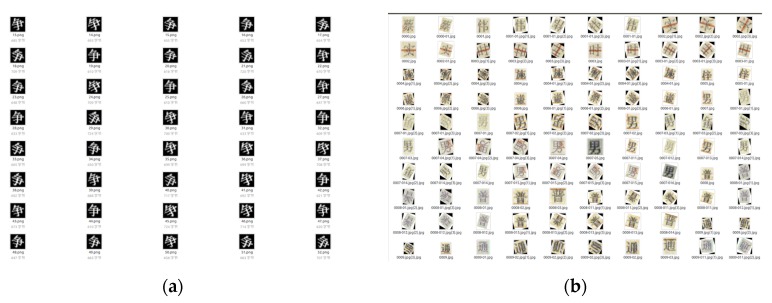
Primary character dataset and self-built dataset. (**a**) Primary character dataset; (**b**) self-built dataset.

Therefore, we simulated the breakpoint effect on the original font according to the principle of the printing breakpoint font, and used them to form a new dataset (self-built dataset), which included 751,000 images—525,700 images from the primary dataset, and 225,300 images from the new dataset (this kind of structure will be explained in Section 4.3.1). It is shown in Figure 5.

To illustrate our figure clearly, we added English translations to help understand the figure. At the left side is an example of the original dataset (https://github.com/AstarLight/CPS-OCR-Engine/blob/master/ocr/gen_printed_char.py). The Chinese character on the image means “content” in English. On the right side is an example of the Chinese characters from our self-built dataset—the meanings of the Chinese characters are “great”, “real”, “time”, “boy”, “modest”, and “normal”. Both datasets are expanded by rotating at different angels.

Finally, as we already know that so many CNN models have been used in many other areas, in order to find an appropriate one, we used some known CNNs, such as Googlenet, Caffenet, and Alexnet, to select a better network that is suitable for our goal.

From the data shown in Table 1, we can see that Alexnet has a higher accuracy when distinguishing the medical invoices; therefore, we chose Alexnet to continue further with our experiment.

During the Alexnet training, any change of the parameters in a layer would result in changes in the following parameters, leading to the network needing to constantly adapt to the new data distribution. It required more parameters to adjust the vector, and it was harder to train a network because of the existence of the nonlinear problems in the activation function of the operation. From what we discussed above, the input data of each layer of the neural network were normalized to the standard normal distribution, which can solve the problems above, reduce the training time of the network significantly, and accelerate the network convergence effectively.

For further improving accuracy and reducing loss, in the second stage, image preprocessing methods such as binarization were added to obtain a small sample dataset, and the training was conducted again.

We used AA-CNN to train the character dataset and the Relu activation function to reduce the computing costs. The Relu equation is shown in Equation (1):

(1) f(x)=max(0,x).

Next, we will explain how the AA-CNN network worked.

**Figure 6:**
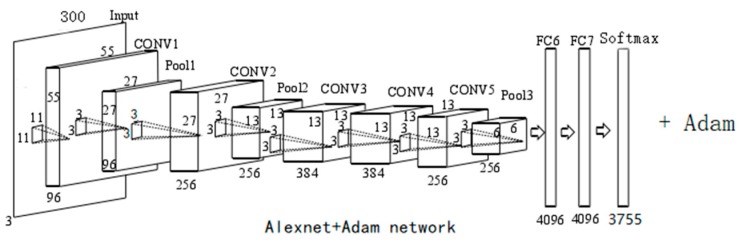
Structure of AA-CNN (Alexnet–Adam–CNN).

As shown in Figure 6, a Chinese character image with a size of 300 × 300 pixels was input into the input layer, and then, after five convolutions (the kernel size of the first convolutional layer was 11 × 11 pixels, while the others’ kernel size was 3 × 3 pixels) and two max-poolings connected to two fully-connected layers, and it finally output 3755 categories—the validation split value was 375,500 and the batch size was 128.

In this article we chose Alexnet in order to make this network more suitable for training the self-built dataset. We made a series of modifications to it, such as the kernel size of the convolutional layer. We also used the Adam optimization algorithm to further reduce the loss value of Alexnet.

The reasons for using AA-CNN for training in this paper were as follows:

1. Local connection and weight sharing: to reduce a large number of parameters, the speed of seal recognition, and classification;
2. Downsampling: to improve the robustness of the model, that is, to improve the accuracy of the medical invoice recognition and model stability;
3. Local response normalization: this normalization was applied after using the nonlinear activation function of ReLu;
4. Overlapping pooling: to reduce the overfitting of images caused by the operation of the non-overlapping adjacent units.

At the same time, three different optimization algorithms were selected based on the Alexnet network, which were as follows:

1. SGD optimization algorithm;
2. Adam optimization algorithm;
3. AdaGrad optimization algorithm.

**Figure 7:**
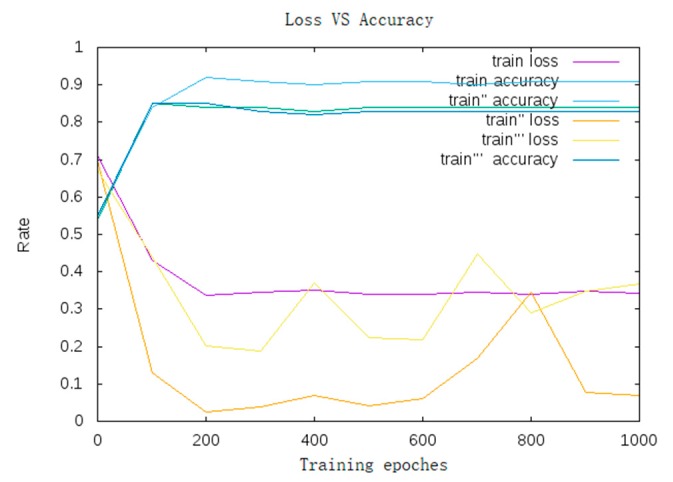
Training curve.

In Figure 7, we can see the Adam optimization algorithm (Adam curve: train “accuracy/train” loss, SGD curve: train accuracy/train loss, AdaGrad curve: train accuracy’’’/train loss’’’)—the loss rate was reduced and the recognition accuracy was improved.

In the procedure of the back propagation, the chain rule was applied on the loss function. The normalized layer was determined by the type of back propagation gradient, including a summation calculation with a small data batch. The calculation equation is shown below:

(2) $$\frac{\partial L}{\partial \sigma^{2}}=-\frac{1}{2}\sum_{i=1}^{s}\frac{\partial L}{\partial g_{i}}\left(e_{i}-\mu \right){\left(\sigma^{2}\right)}^{-\frac{3}{2}};$$

(3) $$\frac{\partial L}{\partial \mu }=\left(\sum_{i=1}^{s}\frac{\partial L}{\partial g_{i}}\frac{-1}{\sigma }\right)+\frac{\partial L}{\partial \sigma^{2}}\frac{-2\sum_{i=1}^{s}(e_{i}-\mu )}{s};$$

(4) $$\frac{\partial L}{\partial e_{i}}=\frac{\partial L}{\partial g_{i}}\frac{1}{\sigma }+\frac{\partial L}{\partial \sigma^{2}}\frac{2\left(e_{i}-\mu \right)}{s}+\frac{1}{s}\frac{\partial L}{\partial \mu }.$$

### 3.2. RNN Model Training for Semantic Revisions

**Figure 8:**
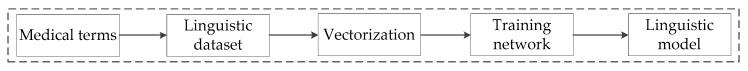
Framework of BPTT-RNN.

This paper introduced the optimization and acceleration of the BPTT-RNN model from the following aspects. The framework is shown in Figure 8.

First, we downloaded the medical terminology from the CNKI (China National Knowledge Infrastructure) and PubMed, making the linguistic dataset include more than 7000 medical terms.

Then, we needed to vectorize the linguistic dataset, preparing for network training.

Finally, in order to obtain the linguistic model, the dataset was applied on the network training.

The RNN was applied to do the semantic revisions using the results of CNN. Then, we gave a brief introduction of BPTT-RNN.

**Figure 9:**
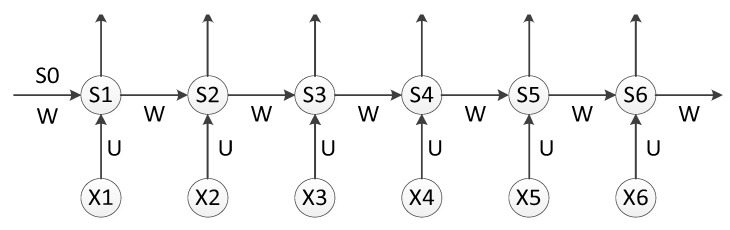
Structure of the circulation layer.

The circulation layer structure is shown in Figure 9.

As is shown in Figure 9, the output of the hidden layer was *o_t_* using *x_t_* as input data. The point is, the value of *s* depended not just on *x_t_*, but on *s_t−1_*. We can use the following equations to express the calculation procedure of cyclic neural network:

(5) $$o_{t}=g\left(V_{s_{t}}\right);$$

(6) $$s_{t}=f\left(U_{x_{t}}+W_{s_{t-1}}\right).$$

Equation (5) is the output layer calculation function. The output layer was a full connection layer. *V* is the weight matrix of the output layer, and *g* is the activation function. Equation (6) is the hidden layer (circulation layer) calculation function. *U* is the weight matrix for the input *x*, *W* is the value for the last time, *s_t−1_* is the input weight matrix for this time, and *f* is the activation function (ReLU).

The BPTT algorithm is a training algorithm for a cyclic layer. Its basic principle is the same as the BP algorithm, and it also contains the same three steps:

1. Forward calculation of each neuron output value;
2. The error term $\delta_{j}$ value of each neuron is calculated reversely. It is the partial derivative of the error function E to the weighted input $net_{j}$ of the neuron *j*;
3. Calculate the gradient of each weight.

Finally, we used the stochastic gradient descent algorithm to update the weight, using the previous Equation (6) to carry out forward calculation for the circulation layer:

Then, we expanded Equation (6) to get Equation (7):

(7) $$\left[\begin{array}{c} s_{1}^{t}\\ \vdots \\ s_{n}^{t} \end{array}\right]=f\left(\left[\begin{array}{ccc} u_{11} & \cdots & u_{1m}\\ \vdots & \ddots & \vdots \\ u_{n1} & \cdots & u_{nm} \end{array}\right]\left[\begin{array}{c} x_{1}\\ \vdots \\ x_{m} \end{array}\right]+\left[\begin{array}{ccc} w_{11} & \cdots & w_{1n}\\ \vdots & \ddots & \vdots \\ w_{n1} & \cdots & w_{nn} \end{array}\right]\left[\begin{array}{c} s_{1}^{t-1}\\ \vdots \\ s_{n}^{t-1} \end{array}\right]\right).$$

The left side of Equation (7) is an output vector, *s_t_*, with a size of *n*.

The right side of Equation (7) has an input vector, *x,* with a size of *m*; the dimension of matrix *U* is n*m; and the dimension of matrix *W* is n*n.

$s_{n}^{t}$ represents the value of the *n*_th_ element of the vector *s* at time *t*, *u_nm_* means the weight from the *m*th neuron in the input layer to the *n*th neuron in the circulation layer, and *w_nn_* means the weight from the *n*th neuron at time *t*−*1* of the circulation layer to the *n*th neuron at time *t* of the circulation layer.

### 3.3. Recognition

**Figure 10:**
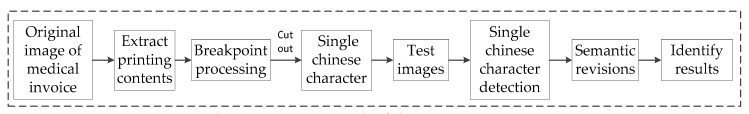
Framework of the recognition part.

After the CNN and RNN model training, the recognition module could carry out the offline operation. The specific procedure is shown in Figure 10.

We got the image of the original medical invoice obtained by a scanner, then extracted the parts we needed to identify through the color threshold.

Finally, extracted text preprocessing (breakpoint processing) was performed. Breakpoint processing is a process with two steps. The first one is Gaussian blur (in this paper, a Gaussian blur with a 0.3 pixel radius was adopted for the medical invoices). The Gaussian blur equations are shown as follows:

(8) $$f\left(x\right)=\frac{1}{\sigma \sqrt{2\pi }}e^{-{\left(x-\mu \right)}^{2}/2\sigma^{2}};$$

(9) $$G\left(x,y\right)=\frac{1}{2\pi \sigma^{2}}e^{-\left(x^{2}+y^{2}\right)/2\sigma^{2}}.$$

In the equations above, σ means the radius of the pixels, the radius of the Gaussian distribution (*σ*) is 0.5, μ is the central point, which is generally zero, and (*x,y*) are the relative coordinates of the peripheral pixels to the center ones. In Equation (8), *f(x)* is the density function of the normal distribution in one dimension, also known as the Gaussian distribution. In Equation (9), G(*x,y*) is the two-dimensional Gaussian equation derived from Equation (8).

After the Gaussian blur processing, the image was further enhanced by a smoothing operation. In order to get a clearer outline (high frequency and intermediate frequency information of the image) of the breakpoint font, we used the mean value filtering to process the image after the Gaussian blur. The equations are shown in Equations (10) and (11):

(10) $$g\left(x,y\right)=\frac{1}{mn}\sum_{\left(x,y\right)\in S_{xy}}f\left(s,t\right);$$

(11) $$g\left(x,y\right)=\frac{\sum_{s=-a}^{a}\sum_{t=-b}^{b}w\left(s,t\right)f\left(x+s,y+t\right)}{\sum_{s=-a}^{a}\sum_{t=-b}^{b}w\left(s,t\right)}.$$

A kernel with the size of m*n (*m*, *n* is odd) was applied on the image as the mean filtering, and the value of the intermediate pixel was replaced by the pixel average of the region covered by the kernel, *g(x, y)* means the center point at *(x, y)* with the size of the m*n filter window, and *f* (*s*,*t*) means the activation function. It is calculated as Equation (10).

For the images of size M*N, the weighted mean filter whose window was the size of M*N is calculated as Equation (11).

## 4. Experimental Results and Analysis

### 4.1. Experimental Settings

The experiments conducted in this paper were as follows: the operating system was Ubuntu 16.04; the GPU was NVIDIA GEFORCE GTX 1060; the memory size was 16 GB; and the software platform was python tensorflow.

**Figure 11:**
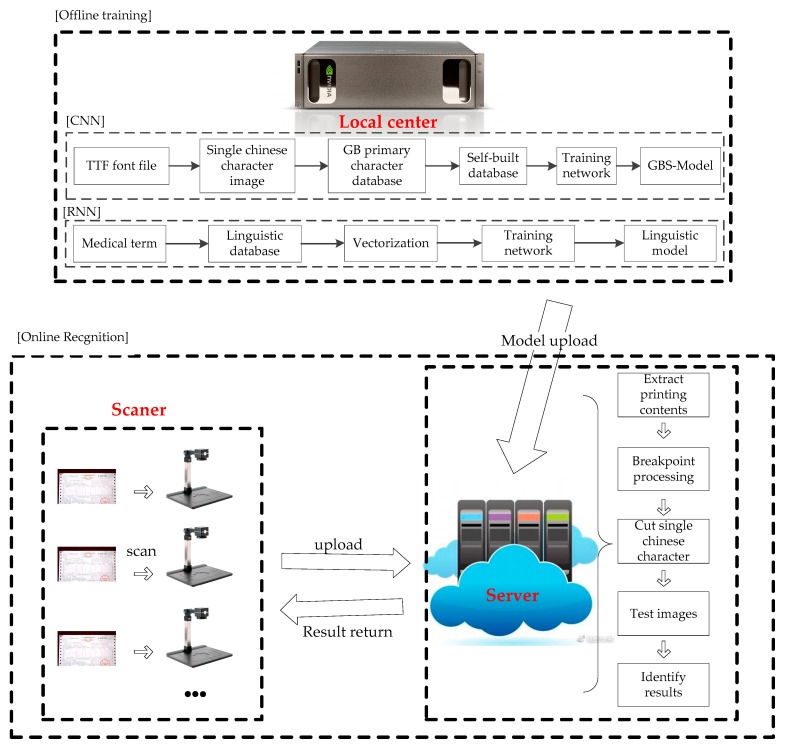
The workflow of the whole system.

The workflow of the whole system is shown in Figure 11.

### 4.2. Comparisons of the Experimental Results

**Figure 12:**
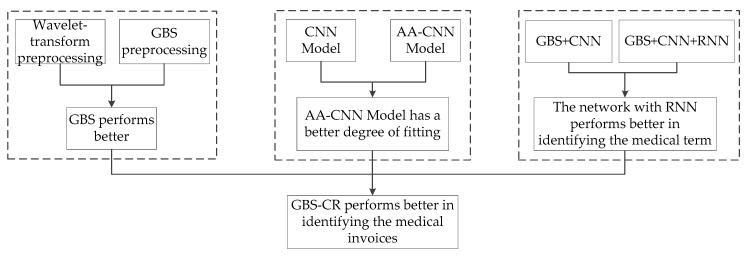
Framework of the experimental comparisons.

In Section 4.2, the experimental results are compared, as shown in Figure 12.

#### 4.2.1. Comparison of Preprocessing

In this paper, in order to achieve a better recognition effect, we used two different preprocessing methods to process the medical invoices, namely:

1. Original preprocessing: wavelet transform based on different wavelet basis functions;
2. GBS preprocessing: Gaussian blur and smoothing processing.

##### Wavelet-Transform Preprocessing

**Figure 13:**
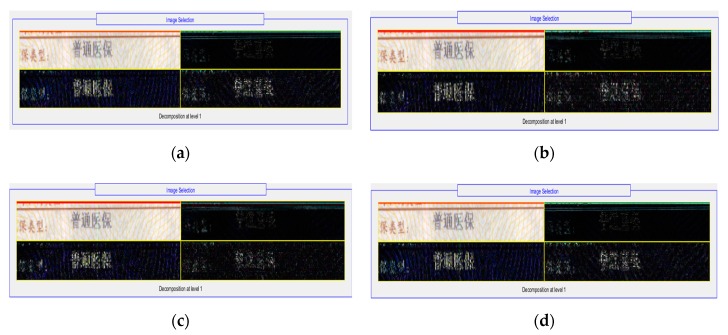
Wavelet transform based on different wavelet basis functions. (**a**) haar; (**b**) db2; (**c**) coif; (**d**) bior.

In traditional image preprocessing, we used wavelet-transform based on different wavelet basis functions to enhance the original image, such as haar, db2, coif, and bior. The results are shown in Figure 13.

Based on the images above, we found that the difference was not obvious between the four kinds of wavelet-transform, so we selected two of them (haar and db2) to continue the following experiment.

**Figure 14:**
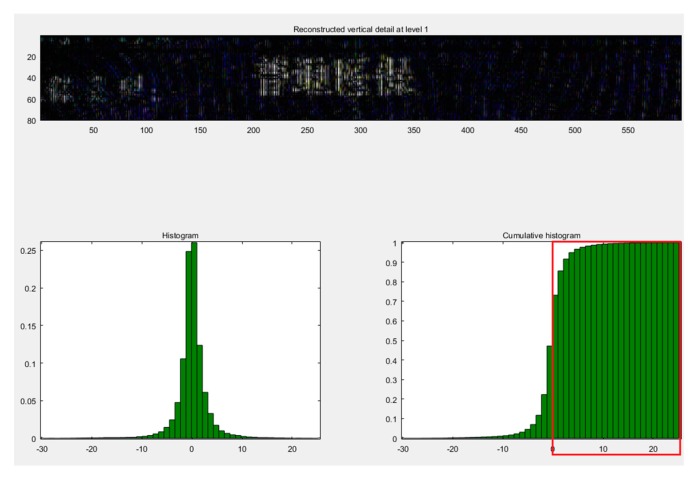
The image using the cumulative statistical histogram after the db2 wavelet-transform.

**Figure 15:**
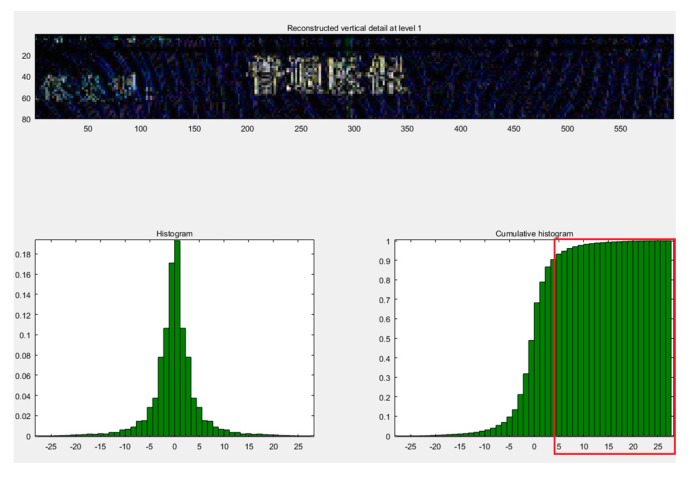
The cumulative statistical histogram of the image after the haar wavelet-transform.

The detailed cumulative histogram images by wavelet-transform based on the haar and db2 wavelet basis functions are shown in the Figure 14 and Figure 15. We observed that the image after the haar wavelet-transform had a more stable peak value from 0 to 25, but wavelet-transform based on the db2 basis function only had a peak value from 0 to 20. So, the wavelet-transform based on the haar basis function was more applicable to this experiment. However, the breakpoint font was still not addressed very well.

##### Breakpoint Processing

**Figure 16:**
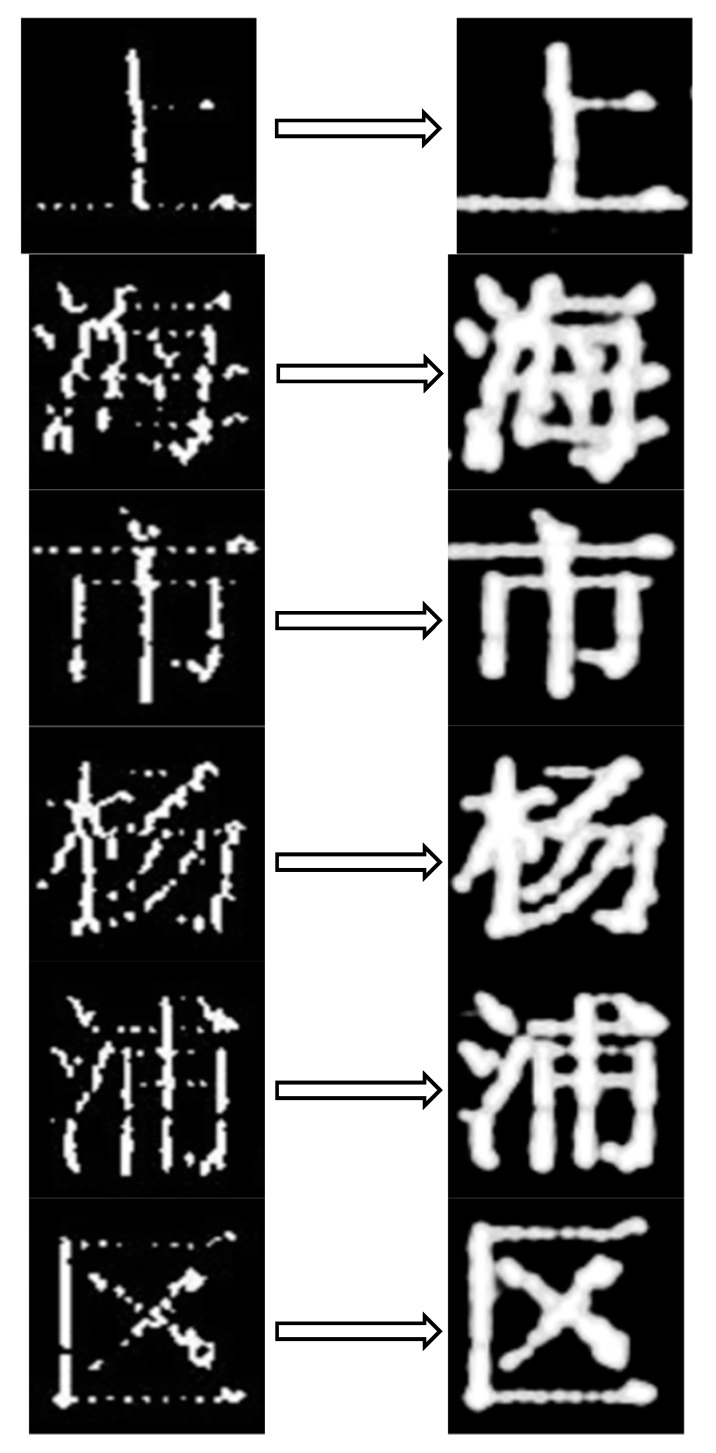
The effect of the character images after GBS preprocessing.

In this paper, a breakpoint processing method was proposed, as shown in Figure 16, based on Gaussian blur and smoothing (GBS).

We found that all of the breakpoint fonts were fixed after GBS preprocessing.

**Figure 17:**
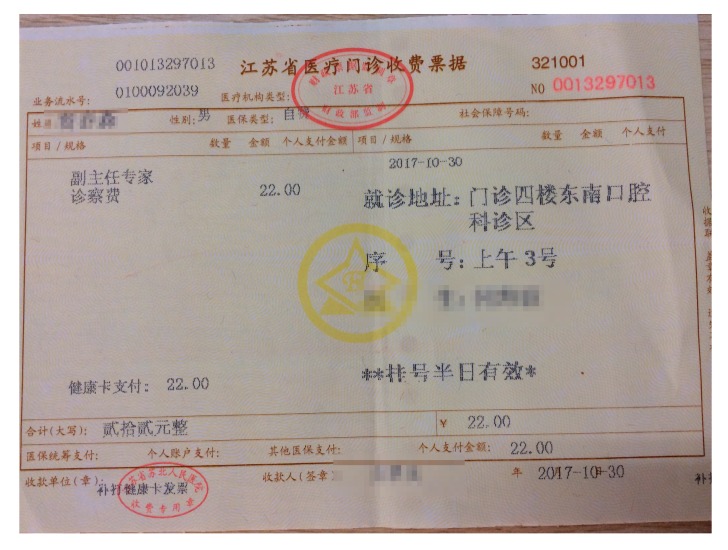
The original medical invoice.

**Figure 18:**
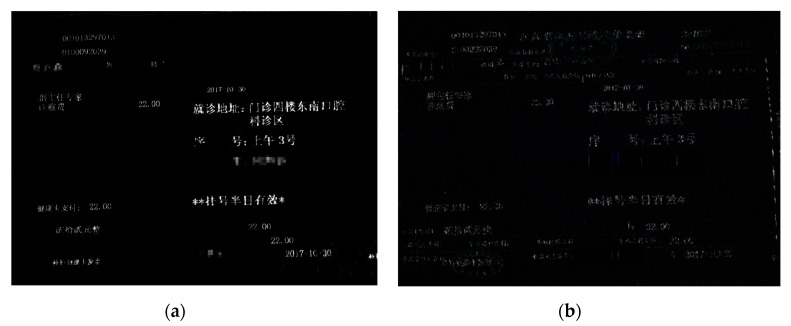
The effect of two preprocessing methods. (**a**) GBS preprocessing; (**b**) wavelet-transform preprocessing.

**Figure 19:**
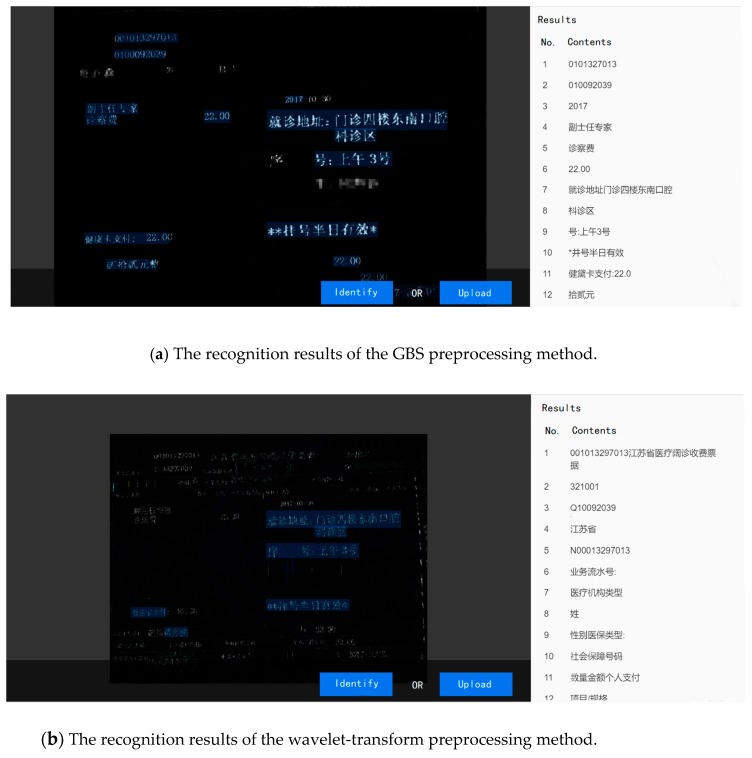
The recognition results of two preprocessing methods.

In order to further confirm the validity of the breakpoint processing, we carried out the following experiments. We selected a medical invoice randomly, and identified it after the wavelet-transform and GBS preprocessing with our program. The whole process is shown in Figure 17, Figure 18 and Figure 19.

In total, there were 157 Chinese characters in this medical invoice. We found that in Figure 19a, 138 Chinese characters were right, while in Figure 19b, only 115 Chinese characters were right. The recognition accuracy of these two methods is shown in Table 2.

From the data in Table 2, it is not hard for us to see that the GBS preprocessing obtained a higher recognition accuracy in this experiment.

#### 4.2.2. Comparison of Normal CNN and AA-CNN

**Figure 20:**
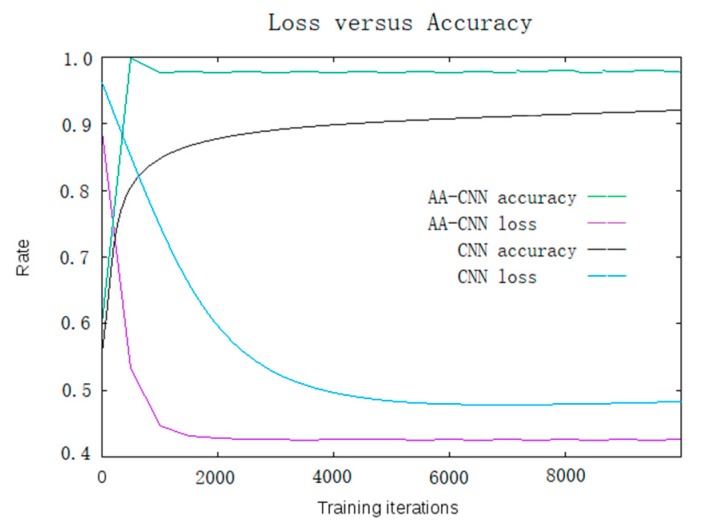
Comparison of the training accuracy and loss between CNN and AA-CNN.

In this section, we will compare the normal CNN with AA-CNN in the same training procedures (training set: 751,000; test set: 37,550; base learning rate: 0.01; iteration: 9000). The training curve is shown in Figure 20.

From Figure 20, we observed that the AA-CNN achieved a higher accuracy and lower loss compared with the normal CNN.

#### 4.2.3. Comparison of Semantic Revisions (RNN)

In this experiment, CNN and RNN were combined to form a parallel computing system. After the CNN recognition result was obtained, RNN was applied to conduct a semantic revision based on the CNN recognition result, which could greatly improve the recognition accuracy of our system for medical invoices.

In order to further confirm the validity of the semantic revisions, we selected a medical invoice randomly, and carried out the following experiments.

**Figure 21:**
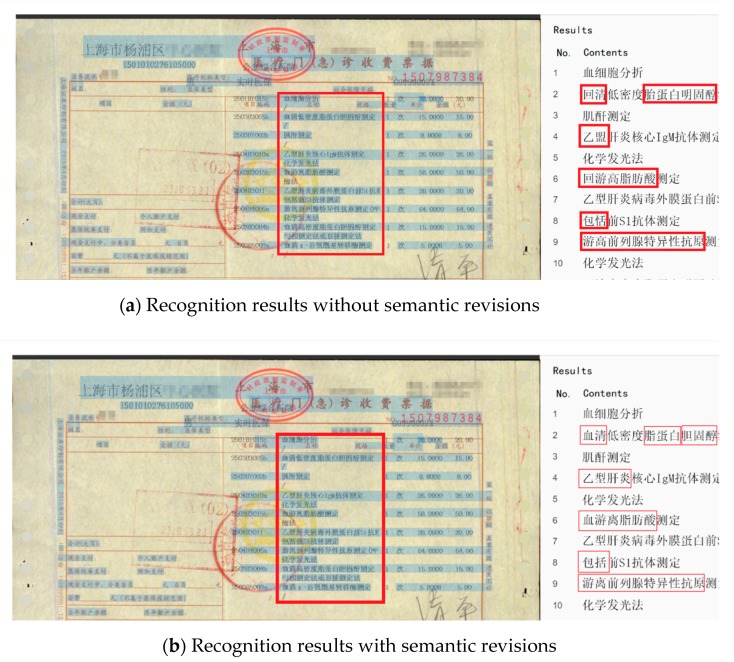
Comparison of the recognition results with or without semantic revisions.

From Figure 21a, we can see that some medical terms were wrongly recognized, such as “serum”, “hepatitis B”, and “nonesterified fatty acid”, which were wrongly recognized in Figure 21a, while in Figure 21b, because of incorporating the semantic revisions module, all of the medical terms were recognized properly.

There were a total of 209 Chinese characters in the selected medical invoice and 175 of them were recognized correctly in Figure 21a, while 191 of them were recognized correctly in Figure 21b. Table 3 shows the accuracy comparison of the recognition results with or without semantic revisions.

As shown in Table 3, as we added a semantic revision module, it examined the recognition results of CNN by medical terminology, so that some characters that were difficult to recognize in CNN could be revised in this module. Thus, the recognition accuracy was higher.

#### 4.2.4. Comparison of Other Machine Learning Techniques

In this experiment, CNN and RNN were combined to form a parallel computing system. In order to further confirm the validity of our method, we selected the same medical invoice used in Section 4.2.3, and carried out the following experiments with HMMs and other DCNN [53,54].

From Table 4, we can see that, compared with other machine learning techniques, the GBS-CR method proposed in this paper still maintained a good performance in recognition. This is because of two main reasons. Firstly, we cited a new preprocessing method (GBS), which could fix the special font in the medical invoices, while the other methods could not. Secondly, after the CNN output its recognition results, we used them as the input of RNN (the semantic revision part) and the RNN gave out the final revised results. In conclusion, there was an average increase of 7 to 12 percentage points in the recognition rate due to our new method.

### 4.3. Experimental Analysis

#### 4.3.1. Analysis of the Dataset

**Figure 22:**
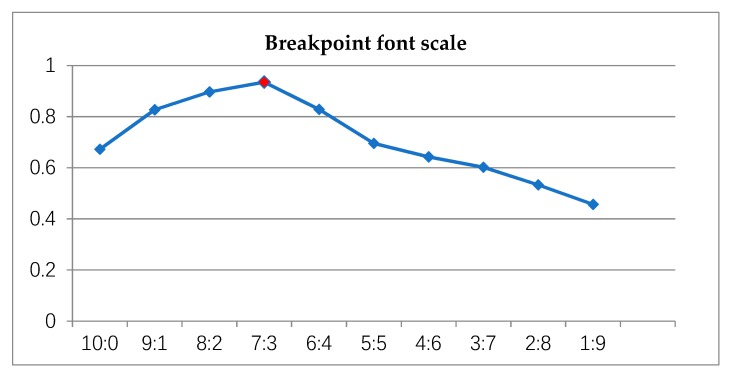
Recognition accuracy under different proportions of breakpoint font.

In this section, we analyzed the dataset in the same training procedures (network: AA-CNN; base learning rate: 0.01; iteration: 8000). We adopted two kinds of datasets, one was the primary font dataset including 3755 commonly used Chinese characters, all of which were continuous, and the other was the self-built dataset mixed with both a continuous and a breakpoint font. Throughout the experiment process, we found that the training accuracy of the model would vary with the different proportions of the breakpoint font, as shown in Figure 22.

It can be seen from Figure 22 that the precision of the model training reached the highest point when the proportion was 7:3 (the font of breakpoint was 3, the original font was 7), while the recognition accuracy was 93.49%.

So, in the other experiments carried out in this paper, we chose this proportion of dataset (self-built dataset).

#### 4.3.2. Analysis of Generalization Ability

In daily life, invoices are not usually kept in a perfect environment. There are a lot of invoices that will be conveniently put in a place, such as in a pocket, and then soaked by water, or rubbed by hands. Therefore, in this section, we focus on the generalization ability of the proposed method, and have made a comparison of the recognition accuracy.

##### Hand-Rubbed Medical Invoices

**Figure 23:**
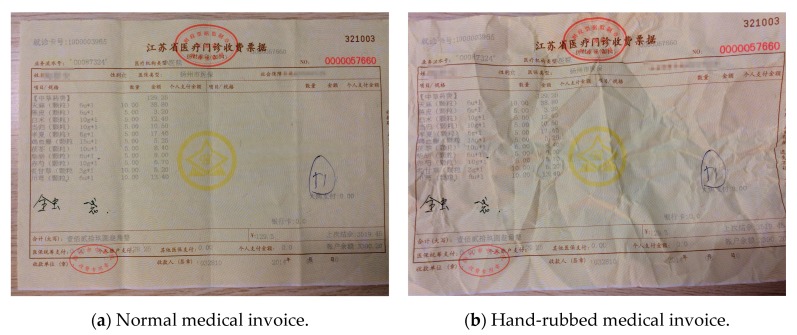
Normal and hand-rubbed medical invoices.

In this part, we selected a medical invoice randomly, and carried out the following experiments. As shown in Figure 23b, it was a hand-rubbed medical invoice, and we did the contrast experiments with the original one in the Figure 23a. We first used the GBS-CR method proposed in this paper to identify the two invoices and to count their accuracy. Then, OCR was used to identify the same hand-rubbed invoice, and its accuracy was compared with the GBS-CR method.

**Figure 24:**
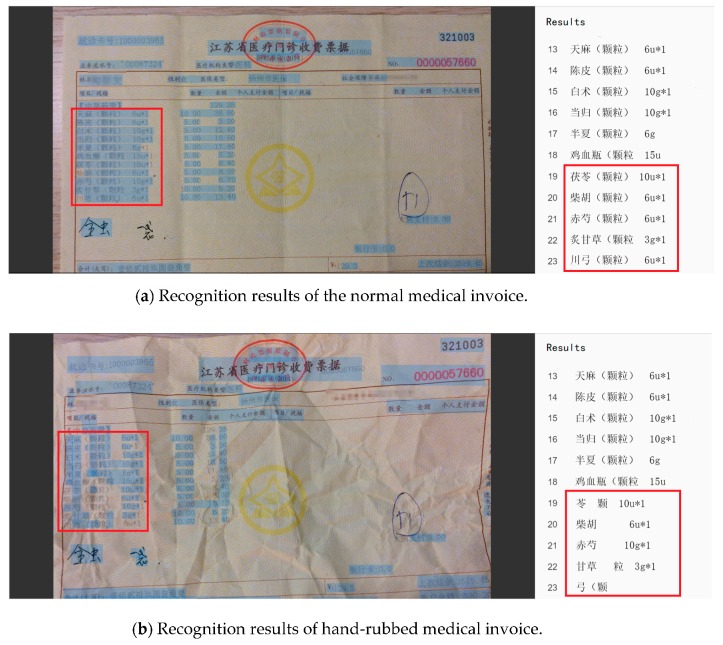
Recognition results of two kinds of medical invoices.

As shown in Figure 24, we used the red box to interpret part of the recognition results. From the comparison of the red box in Figure 24a,b, we observed that some key information was omitted from the hand-rubbed invoice, and some of the results were wrong. According to the statistics, 163 of the 177 characters were correctly identified in the normal medical invoice, while 151 of the 177 characters were correct in the hand-rubbed one. The recognition accuracy is shown in Table 5.

It can be seen from Table 5 that although the invoice had been hand-rubbed, our system (GBS-CR) could still maintain a good performance (only 8 of the 177 characters were omitted or recognized wrongly).

**Figure 25:**
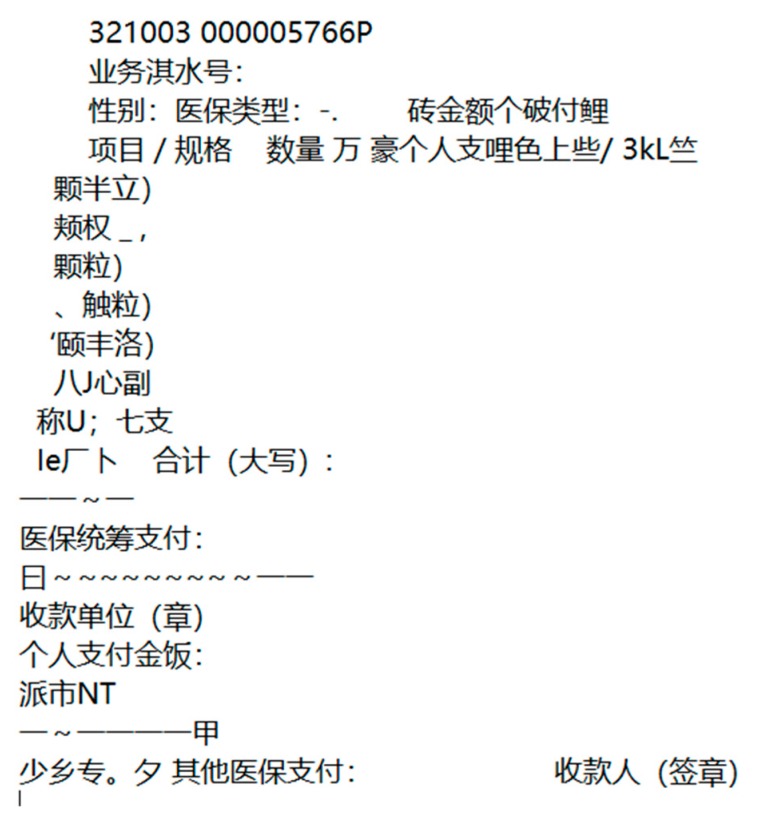
Recognition results of the optical character recognition (OCR) method.

Then, we did the same experiment with another text recognition method called optical character recognition (OCR), and compared the recognition accuracy with our method. According to the statistics, 151 of the 177 characters were correctly identified in the GBS-CR method, while 119 of the 177 characters were correct in OCR (a part of the recognition results is shown in Figure 25).

From Figure 25, we observed that many characters were omitted and even recognized wrongly with the OCR method, and the recognition accuracy is shown in Table 6.

##### Waterlogged Medical Invoices

**Figure 26:**
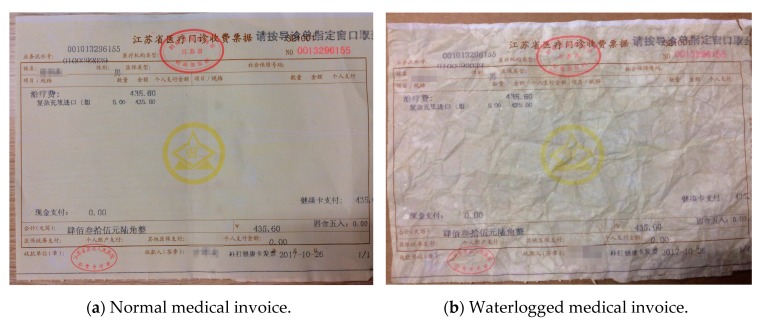
Normal and waterlogged medical invoices.

In this part, we also randomly selected a medical invoice and carried out the following experiments. As shown in Figure 26b, it was a waterlogged medical invoice, and we did contrasting experiments with the original one in Figure 26a. We first used the GBS-CR method proposed in this paper to identify the two invoices and to count their accuracy. Then, OCR was used to identify the same waterlogged invoice, and its accuracy was compared with the GBS-CR method.

**Figure 27:**
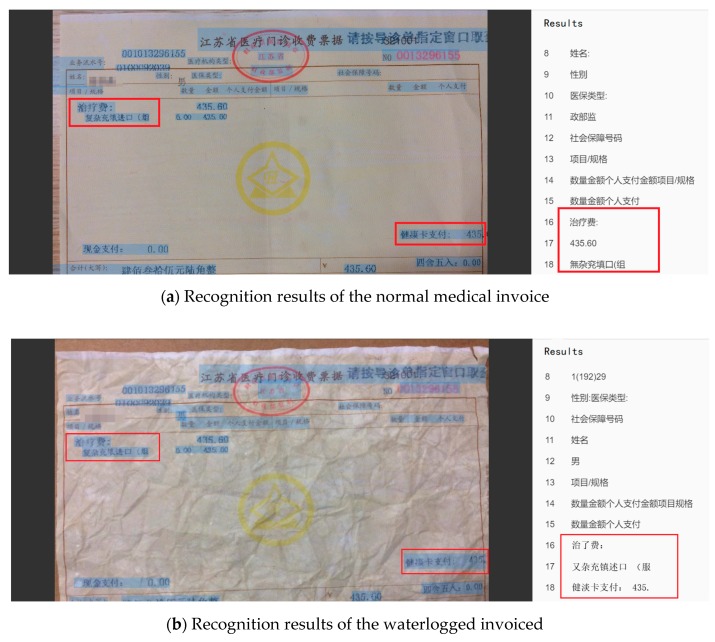
Recognition results of two kinds of medical invoices.

We first used the GBS-CR method proposed in this paper to identify the two invoices and to count their accuracy. The results are shown in Figure 27a,b.

As shown in Figure 27, we also used the red box to interpret part of the recognition results. From the comparison of the red box in Figure 27a,b, we found that just a little information was omitted from the waterlogged invoice; however, some results were recognized wrongly as well. According to the statistics, 116 of the 127 characters were correctly identified in the normal medical invoice, while 106 of the 127 characters were correct in the waterlogged one. The recognition accuracy is shown in Table 7.

It can be seen from Table 7 that although the invoice was waterlogged, our system (GBS-CR) could still maintain a good performance (only 11 of the 127 characters were omitted or recognized wrongly).

**Figure 28:**
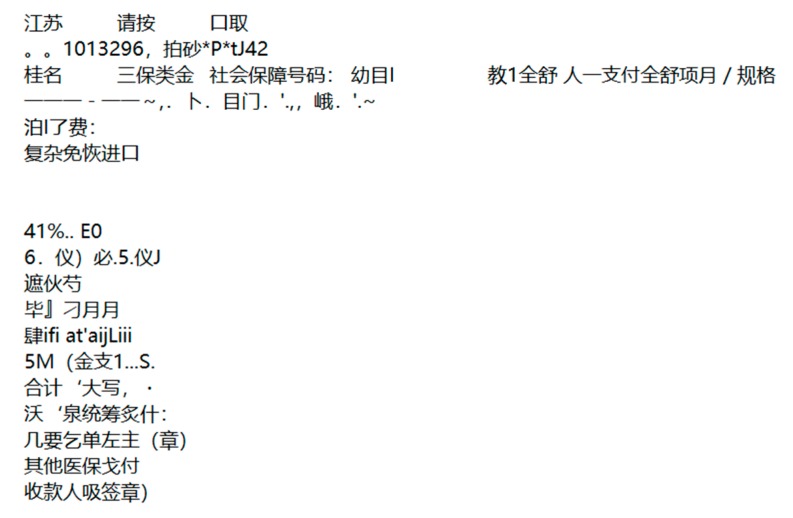
Recognition results of the OCR method.

Then, we did the same experiment with the OCR method, and compared the recognition accuracy with ours. According to the statistics, 83 of the 127 characters were correct with OCR (a part of the recognition results is shown in Figure 28).

From Figure 28, we observed that many characters were omitted and even recognized wrongly with the OCR method, and the recognition accuracy is shown in Table 8.

From Table 8, we can see that, compared with the OCR method, the GBS-CR method proposed in this paper maintained a good performance in the generalization ability, and had an average increase of 10 to 15 percentage points in the recognition rate.

### 4.4. Summary of Experiment Analysis

From the analysis above, we have summarized the following points:

1. By careful design, we adjusted and modified the existing CNN network, and combined it with an Adam optimization algorithm to derive a new CNN network, called AA-CNN;
2. We proposed a novel preprocessing method, consisting of a Gaussian blur and smoothing operation (GBS), in order to fix the breakpoint font. This could improve the recognition accuracy to a certain extent;
3. We designed a medical semantic revision module, which was presented in this paper. It had an average increase of seven to eight percentage points in the recognition rate;
4. We built a new dataset, with an optimal performance comparable to the state-of-the-art method, which used a self-built dataset combining the features of continuous Chinese characters and breakpoint font in medical invoices;
5. In this paper, the generalization ability of our system also had a good performance.

## 5. Conclusions and Future Work

This paper proposed a dual model medical invoice recognition method based on deep learning, which can extract and accurately recognize many breakpoint fonts in real medical invoices, and can solve the problem of the complexity and high error rate of manual classification output in practical applications. In addition, for breakpoint font, a bidirectional approximation method was used to solve the problem of the difficult recognition of the breakpoint font in an impact printer. On the one hand, in the preprocessing section, GBS (Gaussian blur and smoothing) was used to preprocess the collected images of the medical invoices, and the font of the breakpoint can be well recovered. Furthermore, in the training of the AA-CNN model, a self-built dataset with a 7:3 proportion (the font of breakpoint is 3, the original font is 7) of the breakpoint font was used to further improve the recognition accuracy of the system for the typeface of the breakpoint printed by the impact printer. In the process of training the AA-CNN network, a fine-tuned CNN combined with an Adam optimization algorithm was selected for training. Compared with the traditional network, it greatly accelerated the network convergence speed, reduced the loss value, and improved the recognition accuracy. It solved the problem of the low recognition rate of the text recognition methods (OCR) in identifying the breakpoint font in medical invoices. The algorithm designed in this paper achieved a higher recognition accuracy by GBS preprocessing, and the combination of AA-CNN and the semantic revision module based on RNN. Both the theoretical tests and the results of the contrast experiments show that the method proposed in this paper has more advantages in processing the breakpoint font in medical invoices. In the future, we will also focus on improving the preprocessing methods such as restricted Boltzmann machines (RBMs) [55,56,57,58], and applying our approach to more areas like 3D object recognition and fMRI [59,60,61,62].

## Figures and Tables

**Table 1:** Different CNNs and their recognition accuracy.

Convolutional Neural Network (CNN)	Accuracy (%)
Googlenet	81.05
Caffenet	84.25
Alexnet	87.65

**Table 2:** The recognition accuracy of wavelet-transform preprocessing and Gaussian blur and smoothing (GBS) preprocessing.

Preprocessing Mode	Recognition Accuracy (%)
Wavelet-transform preprocessing	73.25
GBS preprocessing	87.91

**Table 3:** The accuracy comparison of the recognition results with or without semantic revisions.

Semantic Comparison	Accuracy (%)
GBS + CNN	83.73
GBS + CNN + RNN	91.39

**Table 4:** Other machine learning techniques and their recognition accuracy compared with GBS-CR.

Other Machine Learning Techniques	Accuracy (%)
GBS-CR	91.39
HMMs	81.05
Caffenet	79.25
Googlenet	84.65

**Table 5:** Recognition accuracy of the normal medical invoice and hand-rubbed one.

Recognition Environment	Accuracy (%)
Normal	92.09
Hand-rubbed	85.31

**Table 6:** Recognition accuracy of the hand-rubbed invoice with different methods. OCR—optical character recognition.

Recognition Methods	Accuracy (%)
OCR	67.19
GBS-CR	85.31

**Table 7:** Recognition accuracy of the waterlogged medical invoices.

Recognition Environment	Accuracy (%)
Normal	91.34
Waterlogged	83.46

**Table 8:** Recognition accuracy of waterlogged invoice with different methods.

Recognition Methods	Accuracy (%)
OCR	65.35
GBS-CR	83.46
